# Compiler Optimizations as a Countermeasure against Side-Channel Analysis in MSP430-Based Devices

**DOI:** 10.3390/s120607994

**Published:** 2012-06-08

**Authors:** Pedro Malagón, Juan-Mariano de Goyeneche, Marina Zapater, José M. Moya, Zorana Banković

**Affiliations:** Escuela Técnica Superior de Ingenieros de Telecomunicación, Universidad Politécnica de Madrid, 28040 Madrid, Spain; E-Mails: goyeneche@die.upm.es (J.-M.G.); marina@die.upm.es (M.Z.); josem@die.upm.es (J.M.M.); zorana@die.upm.es (Z.B.)

**Keywords:** embedded system security, side-channel attacks, hiding countermeasure, compiler optimization, MSP430

## Abstract

Ambient Intelligence (AmI) requires devices everywhere, dynamic and massively distributed networks of low-cost nodes that, among other data, manage private information or control restricted operations. MSP430, a 16-bit microcontroller, is used in WSN platforms, as the TelosB. Physical access to devices cannot be restricted, so attackers consider them a target of their malicious attacks in order to obtain access to the network. Side-channel analysis (SCA) easily exploits leakages from the execution of encryption algorithms that are dependent on critical data to guess the key value. In this paper we present an evaluation framework that facilitates the analysis of the effects of compiler and backend optimizations on the resistance against statistical SCA. We propose an optimization-based software countermeasure that can be used in current low-cost devices to radically increase resistance against statistical SCA, analyzed with the new framework.

## 1. Introduction

The concept of Ambient Intelligence (AmI) provides a vision of the Information Society where the emphasis is on greater user-friendliness, more efficient support services, user-empowerment, and human interaction support.

People are surrounded by intelligent intuitive interfaces that are embedded in all kinds of objects and an environment that is capable of recognizing and responding to the presence of different individuals in a seamless, unobtrusive and often invisible way.

In [1] authors identified five basic technology requirements for AmI, three of them related to Wireless Sensor Networks:

- A seamless mobile/fixed communications infrastructure
- Dynamic and massively distributed device networks
- Dependability and security

According to [2], malicious attacks can even end up ruining businesses based on AmI when the system is put out of operation for some time. Malicious attacks can be active or passive. New architectures for nodes in WSN are being presented everyday, using IEEE802.15.4 or Bluetooth [3]. Privacy and security are critical in AmI applications using WSN, as is the case of medical applications [4] where privacy needs a thorough study [5]. An active attack is a deliberate alteration or destruction of data or creation of false data. A passive attack consists of unauthorized monitoring, but not alteration or destruction of data (e.g., wiretapping).

Traditionally, security engineers have focused on making critical devices unaccessible to attackers and encrypting accessible communication, mainly wireless communication, which is easily accessible to an attacker. Therefore, there are two aspects of security: physical security, related to restricting access to concrete devices with concrete implementations, and logical security, encryption algorithms to protect messages and avoid unauthorized devices to communicate with the system as authorized ones.

In an AmI scenario, devices are everywhere, including wired and wireless nodes. Wireless nodes need to be low-power that minimize energy requirements, which implies a reduction of power supply dimensions (battery, energy harvesting mechanisms) and a reduction in cost and size. Moreover, wireless nodes need to be low-cost, in order to make the whole system affordable to users, as they are massively distributed. Low-cost implies few resources to ensure security, protect the system from tampering or prevent hardware modifications.

AmI applications manage critical information, from confidential personal data to access to restricted areas. Low-cost or low-protected nodes are deployed everywhere, which makes it easy for the attacker to physically access a node of the system and to wirelessly communicate between devices.

In traditional cryptanalysis, encryption is viewed as a black box operation that transforms the plain text into the cipher text using a secret key. Algorithm designers assume that input and output data might be available to attackers, but no other information related to the key is available. Many existing encryption algorithms have no practical known weaknesses, and the only way to unlock the secret key is to try all possible combinations. As a result, as long as the number of combinations is large enough, such that a complete search becomes *de facto* impossible, the encryption algorithm is said to be secure.

However, the encryption algorithm has to be implemented and executed on a real device, which will leak additional information related to the secret key that can be used to determine its value. These attacks, which use additional information leaking from the practical implementation, are also known as side-channel attacks (SCAs). First proposed in 1996 [6], they have been used since then to extract cryptographic key material of symmetric and public key encryption algorithms running on microprocessors, DSPs, FPGAs, ASICs and high performance CPUs from variations in power consumption, time delay or electromagnetic radiation.

In March 2008, researchers from the Chair for Embedded Security of Ruhr University Bochum in Germany presented a complete break of remote key-less entry systems based on the KeeLoq RFID technology [7], widely used for key-less access control, basic in AmI scenarios. Their attack works on all known car and building access control systems that rely on the KeeLoq cipher. Measuring the electric power consumption of a device during encryption, the researchers can extract the manufacturer key from the receivers, which can be regarded as a master key for generating valid keys for the remote controls of one particular manufacturer. It can also be applied to the so-called KeeLoq Code Hopping mode of operation (rolling code) that is widely used for key-less entry systems (cars, garages, buildings, etc.).

In June 2009, a simple power analysis attack (SPA) was reported to break a software implementation of Keeloq with a single power trace [8].

Different software and hardware countermeasures have been proposed to protect devices against SCA. The compiler controls the final implementation used in the execution: assembly instructions, hardware resource used (floating point unit, vector processor), and is critical for software countermeasures. SCA countermeasures must be complementary solutions for Wireless Sensor Networks, in combination with system-wide countermeasures detecting misbehaviors in the system [9]. In [10] authors evaluate a proof-of-concept prototype to study how back-end compilation and optimizations affect to the leaked information of a ×86 processor for timing attacks. Its conclusion is that optimizations introduce asymmetries that can be used by attackers to obtain sensitive data with SCA, so they must be disabled. However, as we have seen previously, wireless nodes in AmI applications need to be low-power and low-cost, so it is important to optimize performance to take out the best of devices, as it is done when defining encryption algorithms and protocols [11].

In order to focus on the implementation, the work described in this article considers a worst case scenario from the designer point of view: samples obtained from simulator without noise, using the same leakage model than the used by the attacker. Although the number of traces for an attack to be successful would be much higher than in this situation, we consider interesting the relative resistance against SCA of different implementations.

In this paper we propose a software countermeasure against statistical SCA based on the use of compiler optimizations. It is a compiler countermeasure against side-channel attacks which can be used in current wireless nodes requiring very little modifications from the software developer point of view.

The rest of the paper is organized as follows: Section 2 introduces the side-channel analysis used and gives an overview on proposed countermeasures. Section 3 explains the countermeasure we propose. Section 4 gives information on the experimental setup used to analyze SCA resistance. Section 5 shows and evaluates the effects measured in the experiment. Finally, the most important conclusions are drawn in Section 6.

## 2. Related Work

Side-channel attacks reveal secret information through the non-invasive observation of the execution of a concrete implementation of an encryption algorithm, rather than mathematical analysis of input-output messages.

**Figure 1.**
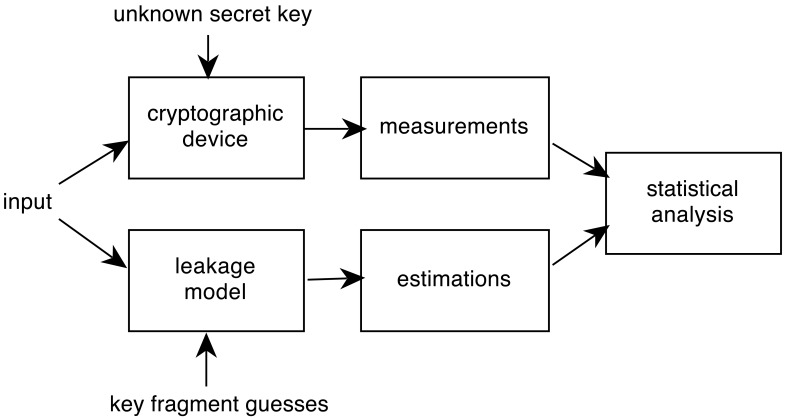
Statistical SCA process.

Statistical analysis against cryptographic devices is one of the most used SCA. There is no need of previous detailed knowledge of the implementation of the algorithm, at the expense of requiring a large number of traces from the device-under-attack. Statistical attacks exploit data dependency of leaked information at fixed moments of time. Figure 1 shows the process followed in statistical attacks.

The most famous statistical attack is Differential Power Analysis (DPA) [12]. The objective of DPA is to recover a subset of the secret key used by the device-under-attack in the encryption of messages. DPA exploits the fact that manipulation of data in standard memory cells and buses leaks information. An attacker detects an intermediate value in the algorithm related to a subset of the key (unknown) and a subset of input or output messages (known) and obtains a large number of measurements. For all the possible values of the subset (reduced when compared to the possible values of the whole key) the intermediate value is calculated, and the information leaked (IL) is estimated, using Hamming Weight (HW) of the intermediate value or Hamming Distance (HD) with a constant value. A threshold for IL is chosen so traces obtained from measurements are assigned to one of two groups, whether IL is greater or lower than the threshold value. An average trace is calculated for each group of measurements and their absolute difference is evaluated. If a number of traces large enough is obtained and chosen input data is distributed uniformly, the difference trace should present a peak in the moment when the intermediate value is manipulated with the correct key and should be close to zero everywhere else.

Correlation Power Analysis (CPA) [13] is another statistical attack which enhances DPA results and reduces the number of traces needed. It shares the first phase with DPA: detect an intermediate value and estimate the information leaked (IL). Instead of grouping traces, CPA requires the evaluation of the correlation coefficient between measured traces and the IL. They are maximally correlated when the intermediate value is manipulated with the correct key.

**Figure 2.**
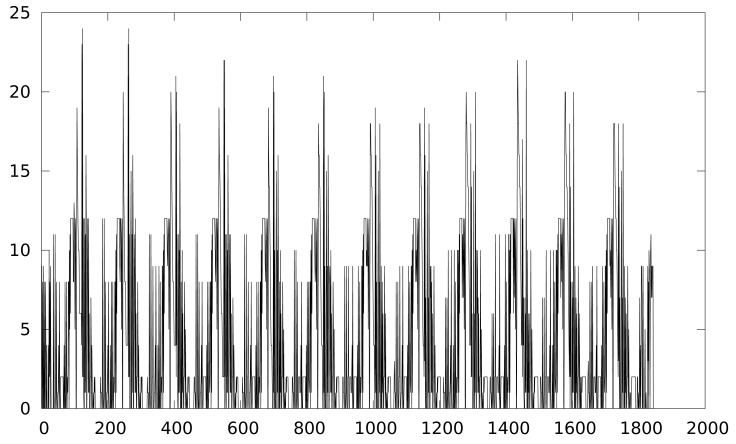
Keeloq power trace (first 12 rounds).

**Figure 3.**
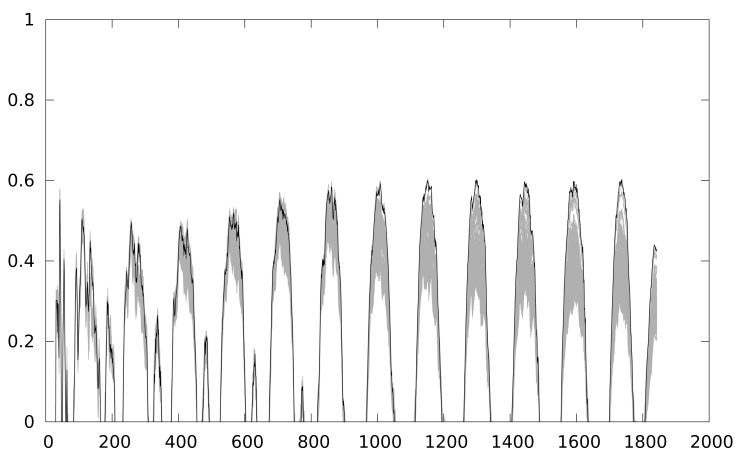
CPA using Keeloq power trace.

Figure 2 shows the leaked information trace while Figure 3 shows the result of a CPA attack applied to a Keeloq implementation executed a MSP430 simulator, which will be described in Section 4. We target the output of the 8-th round, extracting the least significant byte of the key (8 bits), so 256 keys guesses are used in the attack.

Figure 3 depicts that the correlation coefficient has one peak every round, although the attack is focusing the 8-th round. Keeloq is a loop-based algorithm that calculates one new bit every round, so 31 of the 32 bits are similar to the previous round intermediate value. The information leaked in the 8-th round is similar to the 7 previous rounds. The shape of the correlation coefficient is a guarantee that the attack is working properly.

Since SCA first appeared, researchers are evaluating the possibilities of SCA in order to design secure embedded systems. Countermeasures can be both hardware and software. Hardware countermeasures include new logic to avoid information leakage in power consumption [14–16] and randomization units to hide leaked information [17,18]. Software countermeasures include the usage of precomputed tables [19,20] that speeds up execution by increasing memory size and the number of access to memory, which is suitable for AES encryption, but inadequate for Keeloq algorithm.

The increased power attack resistance does not come for free. The algorithmic level masking has a factor 1.5 overhead when compared with a regular (unprotected) design [21]. In [22] authors try to introduce an statistical measure of resistance against SCA, in order to evaluate the convenience of an expensive countermeasure, taking into account that hardware countermeasures can be combined with software countermeasures.

The compiler and its optimizations represent a critical step for software implementations. Software countermeasures inserted by programmers can be changed drastically in this process, introducing asymmetries in final machine code. However, there are few researchers focusing on the compilation and optimization effects.

As we have seen in Section 1, in [10] authors conclude that optimizations need to be disabled to avoid information leakage from software execution, reducing performance. DPA and CPA effectiveness require that the intermediate values are manipulated at the same instant, to achieve appropriate statistical results.

Depending on the optimization applied, the instant when the intermediate value is manipulated is different. Including code compiled with different optimizations and switching between them increases SCA resistance and reduces the vulnerability to Timing Attacks presented in [10] as the reason to disable optimizations. We consider this reduction as variations in execution time between algorithm executions is not only data dependent anymore, but both data and binary code dependent. It is mandatory to select optimizations that change the control flow of the algorithm implementation.

This is a software countermeasure, which implies that analysis using side channel disassembler techniques [23] would still represent a threat. SCARE (SCA Reverse Engineering) is a methodology to exploit side channel information to recover program code. They use Hidden Markov Models to exploit prior knowledge about source code in the target hardware. Different compiler optimizations might generate different source code patterns. On the one hand, they reduce prior knowledge of source code if the optimization sequence is not known. On the other hand, the execution of different implementations can be distinguished with these techniques with a success rate between 58% and 70%, reducing the effect of this countermeasure. However, it is compatible with any hardware countermeasure that reduces success rate of SCARE technique. Moreover, even when the source code is obtained, the complexity of finding the point of interest, where the intermediate value is manipulated, is more difficult. Among compiler optimizations we find obfuscating code transformations [24] that complicates the analysis of binary code.

Regarding statistical attacks, measurements used by researchers to obtain attacks are typically obtained by executing the encryption algorithm in a real device thousands of times, or simulating performance when a new hardware countermeasure is proposed and there is no real device yet. This simulation can be at the logic level, transistor level or layout level. Thousands of measurements are needed because statistical attacks on real devices require thousands of traces to be successful, even in simple devices as MSP430 [25]. In order to gain feedback about the resistance against SCA of a great number of implementations in the same device (applying different combination of optimization passes), this solution is too slow. Moreover, a great knowledge of the device is needed to choose a correct processing of the samples to generate data to compare with the leakage model (peak detector, energy integration in a window). In [26] authors proposed a model to formalize the evaluation of different attacks, over different implementations. They distinguish between an information theoretic metric and a security metric. While the first one should measure the average amount of information that is available in some physical observations, the second one measures how efficiently an actual adversary can turn this information into a successful key recovery.

In our work, we focus on the resistance against SCA of different implementations. Following the recommendations proposed in [26] we assume a very high-skilled adversary who uses a leakage model that maps exactly with the obtained traces.

## 3. Proposed Solution

On the one hand, existing hardware countermeasures require the design and fabrication of new logic that is expensive in area and in energy consumption. On the other hand, software countermeasures seem to be affordable for development, although they need effort from developers in creating countermeasures for each algorithm, and the result is unpredictable due to the presence of a compiler that maps high-level implementations (may be secure) to final machine code, introducing leakages in the process.

We propose the use of optimization chains to create compiler countermeasures against side-channel analysis. Currently, when programming a device, software developers choose one chain of optimizations (traditionally focusing on speed, memory, code). We propose that software developers mark functions with a pragma directive indicating that critical data is being manipulated with other data inside that function. Then, instead of choosing just one optimization chain, it must choose a number of them (3 is the default value), changing small parameters of configuration.

The compiler, when detecting this pragma directive, generates different machine code functions until it gets the specified number of different versions (3 is the default), and randomizes the access to each of them in execution.

The benefits of our solution are:

- Ease of use for developers
- Ease of integration in current solutions
- Improvement of performance when compared to solutions that disable optimizations

Why does this solution improve resistance against side-channel analysis? Our software countermeasure proposal introduces asymmetries in two ways. First, software optimization might introduce conditional sentences. Depending on the value of the key, the manipulation of the intermediatevalue is done in different instants. Traditionally this has been considered as a leakage of information for simple power analysis but a countermeasure for statistical power analysis. Second, using three different implementations that are executed randomly, the attacker cannot associate a deviation in time to a concrete implementation, reducing the impact of deviations in simple power analysis.

Moreover, we could think that a possible solution would be to have a number of completely different implementations of the algorithm randomly executed. Although it seems to be a good approach, big differences in the power consumption pattern would give an attacker a clue about which implementation is being used. When implementations are closely related, with small differences (changing an optimization parameter, disabling an optimization pass in an optimization chain) an attacker cannot recognize the implementation used with visual inspection of the power trace.

## 4. Experimental Setup

Side-channel analysis requires the attacker to measure any magnitude where information from the execution of an encryption secret key might be leaked. Power consumption [12], electromagnetic radiation (EMA) [27], execution time [28], fault injection behavior [29] or cache access [30] are known side-channels.

We focus our experiments on CPA, which can be applied to power consumption traces or to EMA. The goal of our experiment is to improve resistance against SCA using compiler optimizations. Instead of using a real device in our scenario, we model information leaked from the execution of the program in a cycle accurate simulator.

The processor we simulate is MSP430. The MSP430 is the microcontroller used in TelosB [31], a platform traditionally used in WSN. We use an optimization oriented compiler to generate a binary program from the encryption algorithm C program. The simulator used is MSPSim [32] that integrates the radio interface in the simulation as events. MSPSim is used for the simulation of WSN applications that used TelosB platform with COOJA. MSPSim is an instruction accurate simulator, in which register and memory values are available. It has an energy profiling extension for energy consumption estimation, important in the field of WSN and AmI. However, the precision needed power SCA requires the modification of the simulator. Our modification converts the simulator from instruction accurate to cycle accurate, a required approach for power and timing SCA. In the MSP430, every instruction is composed of a maximum of six phases: instruction fetch, decode, source data fetch, destination data fetch, execution and write-back. Not every instruction requires the six phases, and the number of phases executed depends on the addressing mode and the instruction.

The extension we have added to MSPSim takes into account the data loaded to the internal buses of the microcontroller in different instants inside the instruction cycle. Thus, we obtain four different values, using the Hamming Weight model: address bus, register bus, data bus and ALU result. An adversarial might obtain similar values from real devices with expertise, using EM-probes.

A binary program for MSP430 is obtained by compiling from the LLVM binary to the MSP430 target (concretely, we use MSP430F5438A) with LLVM Compiler (llc), which generates MSP430 assembly code. MSP430 assembly code is translated to binary code with msp430 binutils available in Ubuntudistribution (version 2.21). The same binary program can be executed in a real platform and in the MSPSim simulator.

**Figure 4.**
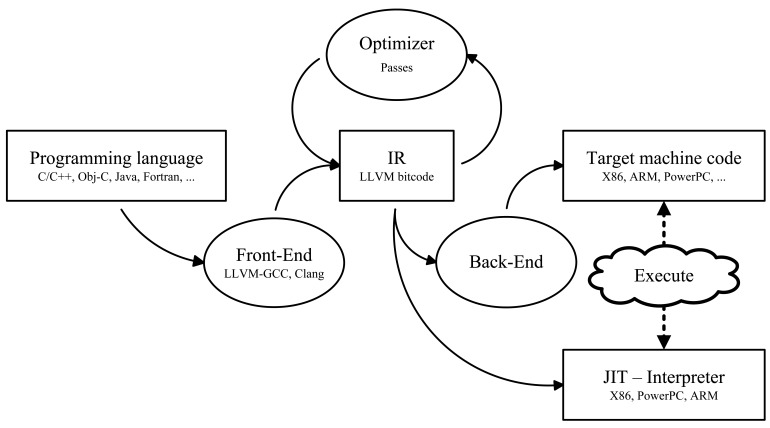
LLVM compilation flow.

LLVM is a Low Level Virtual Machine first created in [33]. It is an optimization centered compiler. Figure 4 depicts the process of compiling with LLVM, where front-end and back-end are decoupled. The front-end compiles from high-level language (C, Java) to an Intermediate Representation (IR). IR in LLVM is LLVM bitcode, and it is executable in the Virtual Machine, with lli, the LLVM interpreter. Over the LLVM bitcode, optimization passes are applied one after the other to generate an optimized bitcode. Optimized code can be executed with lli or compiled to generate assembly code for a target, including processors traditionally used in WSN for AmI applications, such as MSP430 (our case), ARM7TDMI or PIC16.

This solution provides some advantages when compared to implementations in real devices:

- The setup is very simple, avoiding the use of oscilloscopes or EM probes
- Traces are perfectly synchronized and there is no clock deviation
- Time used for setup is drastically reduced (no oscilloscope, no binary loader, no trigger, *etc.*)
- Trace is expected to be better correlated with information leaked, as effects of other peripherals are eliminated

This solution keeps realistic time footprint of the instructions executed in the encryption algorithm. Therefore, resistance against power and timing analysis can be done with this framework. We have extracted the common structure of all the executions performed in our setups, which includes the first 12 rounds of the Keeloq encryption process.

**Figure 5.**
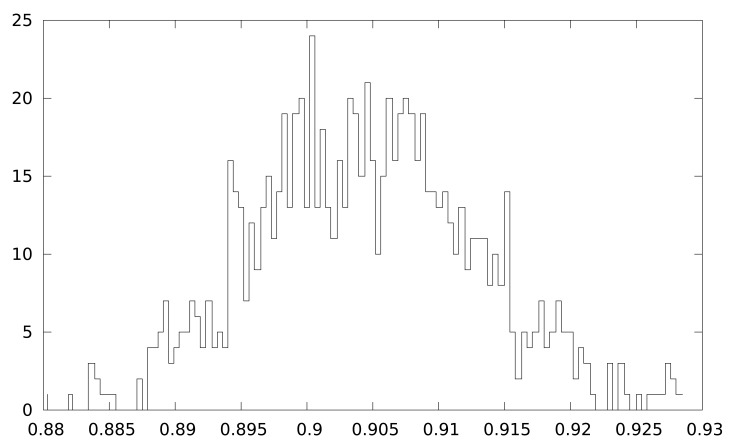
Number of samples with a similar percentual deviation from common code.

Figure 5 depicts the deviation of the program flow from the common code. We consider common code the cycles that have been executed in every measurement, excluding the initialization process (only the encryption algorithm). The percentage of common code in our measurements varies from 88% to 92%, and the deviation depends only on data executed. The distribution shown in Figure 5 follows a normal distribution because data used as input is uniformly distributed.

Moreover, we modify the LLVM interpreter to model information leaked from data and address buses and from register values in LLVM, obtaining an even simpler solution.

This solution provides some advantages when compared to cycle accurate simulator:

- The measurements are even faster
- Only optimization effects are taken into account, avoiding the back-end process, which might change implementation
- Trace is expected to be more correlated with information leaked, as there is no timing deviation depending on data as the number of registers available is infinite
- This is the worst case from the defender's point of view. A small improvement in this scenario might be a large one in real targets.

The main drawback related to a solution based on the LLVM interpreter model is that it does not take into account effects introduced by the backend. LLVM is a 32-bit virtual machine, while most of the microcontrollers used in WSN are 8-bit (PIC16, PIC18 or ATMEGA) or 16-bit (MSP430). In the case of timing or template attacks, this solution is not valid, as the back-end effect on the implementation is critical.

For the experiments, we use a software implementation of Keeloq algorithm, and the LLVM optimization chain std-compile-opts. The main function of the C code is listed, a loop of 528 iterations.

We consider using register information only to increase the efficiency of the attack, as the intermediate value is always loaded into registers. A simpler model, which is traditionally used for simulation of power consumption in processors, would be to take into account the effect of data and address buses, which are typically the longest lines in real devices, and produce the greatest electromagnetic radiations and power consumptions. However, the use of compiler optimizations might reduce access to memory, as the mem2reg optimization pass does, by using registers for intermediate values of the Keeloq loop. The model we use to simulate leakage of the system is the Hamming Weight of the register bus where data is written to in any ALU, shift or load from memory operation.


u_int32_t
KeeLoq_Encrypt (const u_int32_t data, const u_int64_t key)
{
 u_int32_t x = data, r, i;
 for (r = 0; r < 528; r++)
 {
  i = bit (x, 1) + (bit (x, 9) ≪ 1)
   + (bit (x, 20) ≪ 2) + (bit (x, 26) ≪ 3)
   + (bit (x, 31) ≪ 4);
  x = (x≫1) ˆ ((bit (x, 0) ˆ bit (x, 16)
   ˆ (u_int32_t) bit (key, r&63) ˆ bit (KeeLoq_NLF, i)) ≪31);
 }
 return x;
}

In order to compare the feasibility of the attacks in both setups we evaluate the resistance against CPA of a Keeloq implementation with std-compile-opts optimization. The resistance is measured by performing CPA attacks with an increasing number of samples, until the correlation value of the key used for samples stands out.

An attack is successful when the correlation coefficient of the correct key stands out from the rest. Therefore, we evaluate the response of an implementation against CPA as the number of power traces needed to obtain an outstanding correlation coefficient. In order to know the number of power traces needed, we perform several attacks, increasing the number of traces used in each attack.

**Figure 6.**
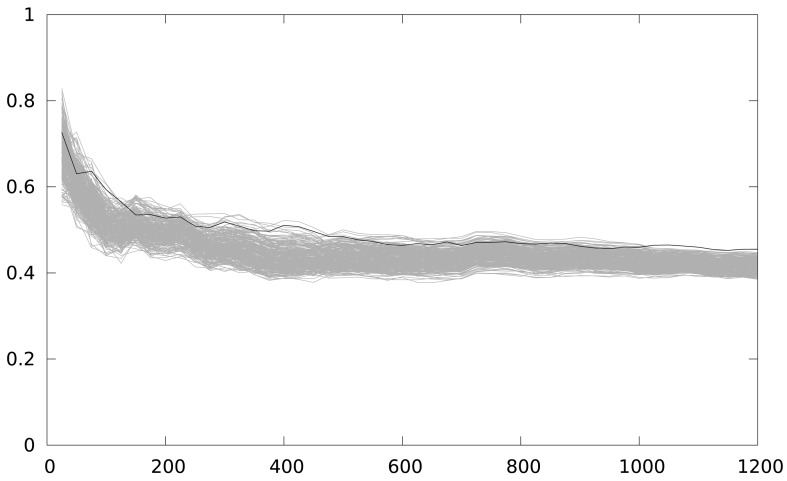
Maximum correlation for different key guesses *vs.* number of power traces with a partial loop unrolling of 2 iterations with MSPSim.

In Figure 6 the number of traces used is in the x-axis. The maximum correlation coefficient obtained is in the y-axis. A Keeloq implementation with a partial loop unrolling of 2 iterations (two iterations form the loop body implementation) is the target of the attack. As we are focusing on an intermediate value of 8 bits, we have 256 candidates, and only one is a correct key guess (the black line), while grey lines (255 lines) are the incorrect key guesses. The figure shows 48 attacks, starting from a 25-power trace, and increasing by 25 the number of power traces used in the attack, until 1,200 samples are used. The number of samples used is indicated in the x-axis.

**Figure 7.**
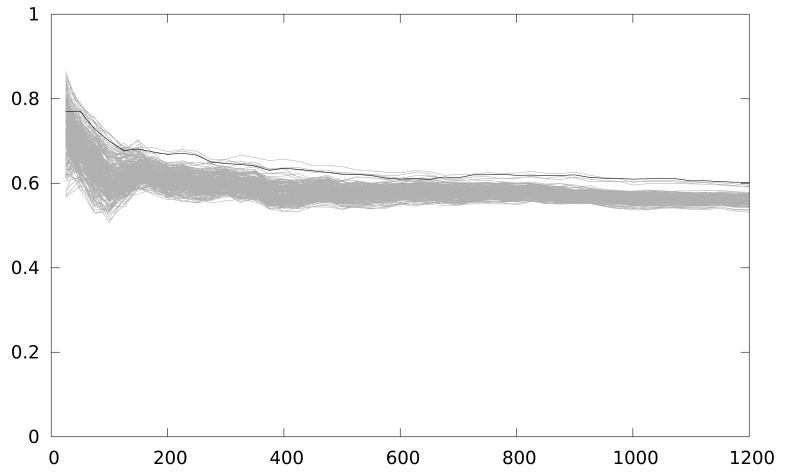
Maximum correlation for different key guesses *vs.* number of power traces with a partial loop unrolling of 2 iterations with MSPSim with 5 cycle window integration.

The correct key guess does not stand out from the rest, as it should be in a completely successful attack, although it is the one with the higher correlation coefficient. We perform an windowed integration in time, to reduce the timing effect of the back-end in the attack. Figure 7 depicts the result of this new attack. The correlation coefficient is higher, and the group of outstanding key guesses is reduced to 4. However, in order to have a deciding result, this solution seems to require a much higher number of samples, which is not practical for analyzing several implementations.

**Figure 8.**
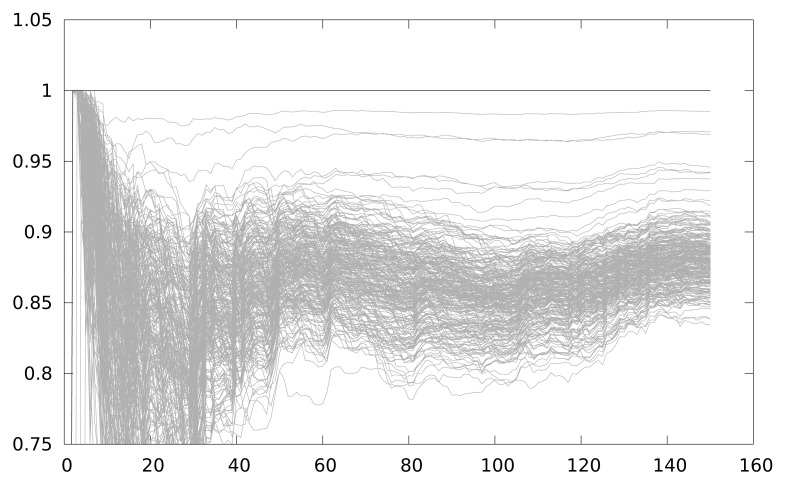
Maximum correlation for different key guesses *vs.* number of power traces with a partial loop unrolling of 2 iterations.

On the other hand Figure 8 depicts the result using the LLVM interpreter model. The figure shows 150 attacks, starting from a 1-power trace attack, and increasing by 1 the number of power traces used every new attack. As seen, the correlation for the correct key guess is always maximum because the power model of the attack simulator corresponds exactly with the attacker power model, and also the power traces are always perfectly aligned. This will not be the case in a real attack scenario, but it is clearly a worst-case scenario for testing countermeasures against DPA attacks.

We use the LLVM interpreter model for the results, leaving the MSPSim model for more accuracy, for timing attacks based experiments or for application integrated attacks.

The compilation chain contains an optimization pass of loop unrolling, which reduces iterations of a loop copying the source code. In the compilation process we only change the number of iterations to unroll, obtaining different machine codes: no loop unrolling, 2-iteration unrolling (loop reduces to 264 iterations) and 3-iteration unrolling.

In the experiment we compare the number of traces needed to guess the correct key when one of these implementations is executed (any of them) and when, at each iteration of the loop, one of the three implementation is randomly chosen to be executed.

## 5. Results

Figure 8 depicts the resistance against CPA of a 2-iteration unrolling implementation. The correct key guess is distinguishable from the rest even using 5 samples. When the partial loop unrolling is of 3 iterations, and when no loop unrolling is applied, in both cases the results obtained are similar. Basically, when one implementation that operates the intermediate value in the same instant is executed, CPA obtains the key with few power traces. This is the current situation in any AmI application using WSN.

**Figure 9.**
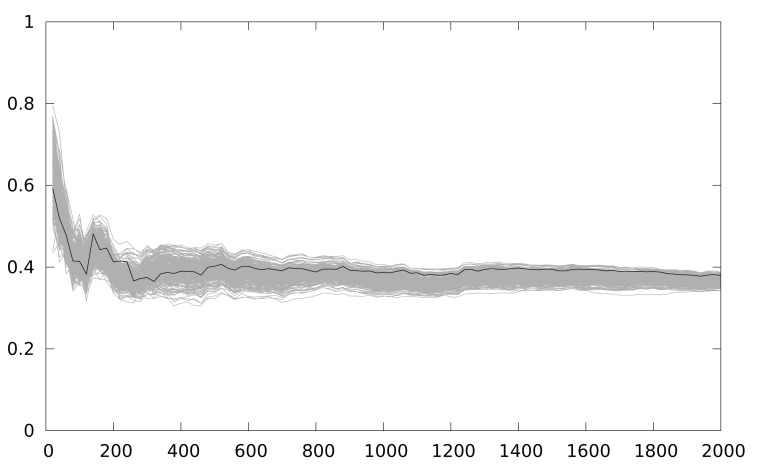
Maximum correlation for different key guesses *vs.* number of power traces when switching randomly between 3 implementations (2, 3 iterations and no unrolling).

Figure 9 shows the result of the second experiment, where our solution has been put into practice. The results are obtained from 100 attacks, starting with 20 power traces, and stepping by 20. We randomly switch between 3 implementations (with 2, 3 iterations and with no unrolling). Using the same attack, it becomes impossible to discriminate the correct key guess even for 2,000 power traces.

**Figure 10.**
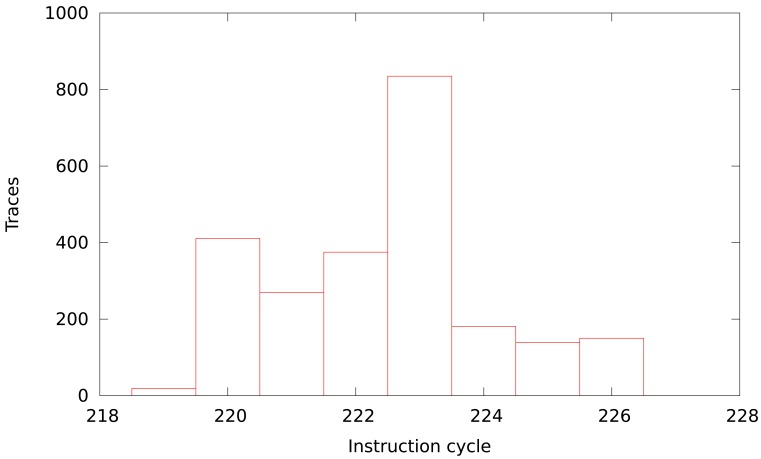
Histogram of manipulation of intermediate value.

Figure 10 depicts the dispersion of the instant when the intermediate value is obtained. This dispersion is the cause of the improvement.

**Figure 11.**
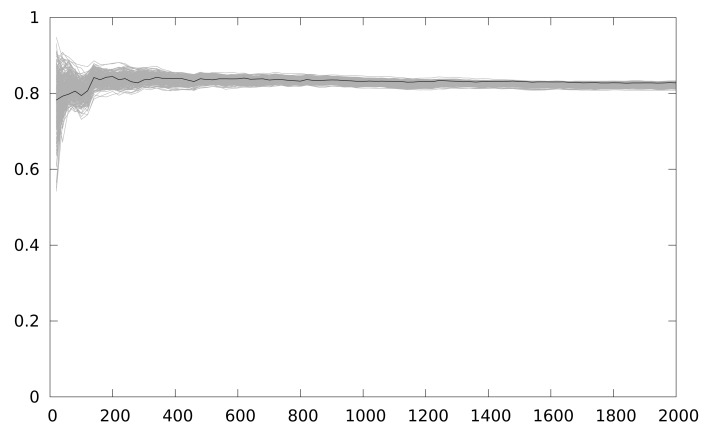
Maximum correlation for different key guesses *vs.* number of power traces when switching randomly between 3 implementations (2, 3 iterations and no unrolling) attacking with window of size 10.

**Figure 12.**
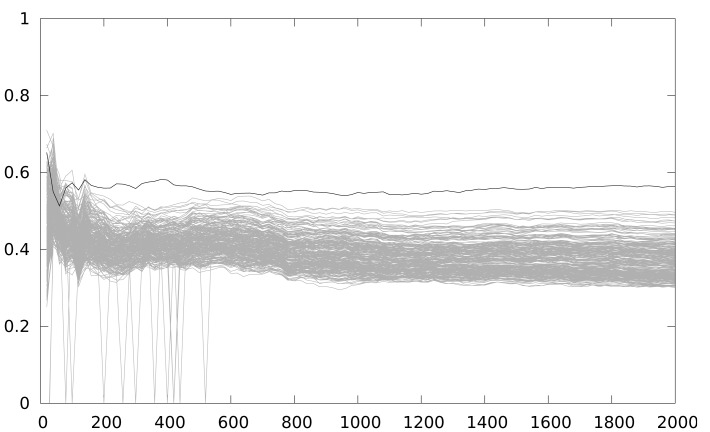
Maximum correlation in region of interest for different key guesses *vs.* number of power traces when switching randomly between 3 implementations (2, 3 iterations and no unrolling) attacking with window of size 10.

**Figure 13.**
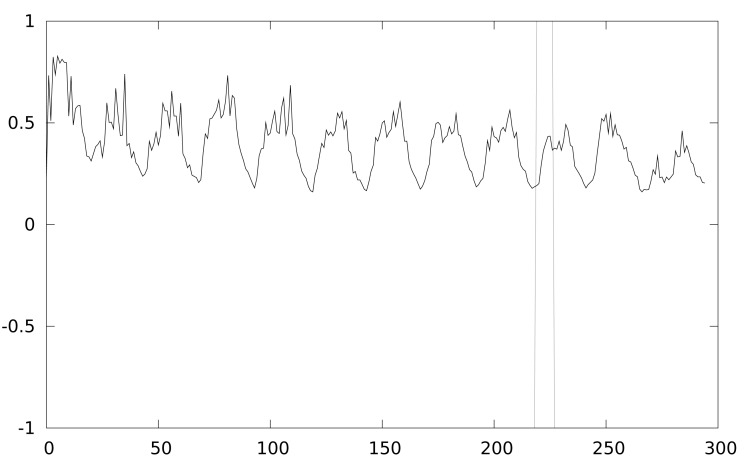
Correlation trace for correct key guess using windowed CPA.

If we improve the attack using a 10 instruction cycle window integration, to make sure the intermediate value is manipulated in that window, correlation values should show better results of the attack. Surprisingly, Figure 11 does not show any improvement. However, if we only take into account the maximum correlation value of the temporal region of interest (between instruction cycles 200 and 250) the key guess is the correct one, as shown in Figure 12. The explanation is that window integration has introduced parasitic correlations (ghost peaks). If the attacker has no knowledge about the implementation of the algorithm, he can be misled by these spurious correlations, as can be seen in Figure 13.

## 6. Conclusions

Side-channel analysis is a threat for AmI applications, where devices are everywhere and any attacker can monitor the leakage from a lot of devices in a network.

We have developed a framework for evaluating the resistance against SCA of different implementations of the same algorithm. The framework is based on LLVM, an optimization-based compiler. It is based on modeled information leakage, with the Hamming Weight of data processed in the execution. A cycle accurate simulator for MSP430 provides realistic information leakage in the power and time domains, adequate for any kind of side-channel attack (even fault injection attacks). The drawback of this solution, when considering the effect of compiler optimizations, are the asymmetries introduced by the compiler back-end generating the code for a real platform with limited resources. Therefore, the framework provides a second leakage model of the implementation, by executing the algorithm with the LLVM interpreter. The latter solution requires fewer samples to guarantee the success of an attack on an unprotected implementation, reducing effects that complicates the setup of an attack.

We have designed a very simple countermeasure against side-channel attacks that enables the use of compiler optimizations to improve performance. In particular, it is well suited for enhancing attack resistance in embedded systems.

The experimental results show at least three orders of magnitude improvement in DPA resistance for a custom Keeloq implementation, by using only static techniques.

Moreover, our approach to avoid side-channel attacks allows a high degree of decoupling between the application development and the security-aware implementation, taking into account compilation, and run-time issues.

Compared with previous countermeasures, our proposal is trivial to integrate in current microprocessor designs, as it has been already integrated in MSP430, and offers a high resistance against side-channel attacks, while maintaining similar power consumption.
